# Molecular Epidemiology and Risk Factors of Carbapenemase-Producing *Enterobacteriaceae* Isolates in Portuguese Hospitals: Results From European Survey on Carbapenemase-Producing *Enterobacteriaceae* (EuSCAPE)

**DOI:** 10.3389/fmicb.2018.02834

**Published:** 2018-11-27

**Authors:** Vera Manageiro, Raquel Romão, Inês Barata Moura, Daniel A. Sampaio, Luís Vieira, Eugénia Ferreira, the Network EuSCAPE-Portugal, Manuela Caniça

**Affiliations:** ^1^National Reference Laboratory of Antibiotic Resistances and Healthcare Associated Infections, Department of Infectious Diseases, National Institute of Health Dr. Ricardo Jorge, Lisbon, Portugal; ^2^Centre for the Studies of Animal Science, Institute of Agrarian and Agri-Food Sciences and Technologies, University of Oporto, Oporto, Portugal; ^3^Innovation and Technology Unit, Department of Human Genetics, National Institute of Health Dr. Ricardo Jorge, Lisbon, Portugal

**Keywords:** carbapenemase-producing *Enterobacteriaceae*, KPC-21, EuSCAPE, Portugal, *Klebsiella pneumoniae*, *Escherichia coli*

## Abstract

In Portugal, the epidemiological stage for the spread of carbapenemase-producing *Enterobacteriaceae* (CPE) increased from sporadic isolates or single hospital clones (2010–2013), to hospital outbreaks, later. Here we report data from a 6-month study performed under the European Survey on Carbapenemase-Producing *Enterobacteriaceae* (EuSCAPE). During the study period, 67 isolates (61 *Klebsiella pneumoniae* and 6 *Escherichia coli*) non-susceptible to carbapenems were identified in participant hospital laboratories. We detected 37 *bla*_KPC–type_ (including one new variant: *bla*_KPC–21_), 1 *bla*_GES–5_, and 1 *bla*_GES–6_ plus *bla*_KPC–3_, alone or in combination with other *bla* genes. Bioinformatics analysis of the KPC-21-producing *E. coli* identified the new variant *bla*_KPC–21_ in a 12,748 bp length plasmid. The *bla*_KPC–21_ gene was harbored on a non-Tn*4401* element, presenting upstream a partial IS*Kpn6* (ΔIS*Kpn6/*Δ*traN*) with the related left IR (IR_L_) and downstream a truncated Tn3 transposon. PFGE and MLST analysis showed an important diversity, as isolates belonged to distinct PFGE and STs profiles. In this study, we highlighted the presence of the high-risk clone *E. coli* sequence-type (ST) 131 clade C/H30. This worldwide disseminated *E. coli* lineage was already detected in Portugal among other antibiotic resistance reservoirs. This study highlights the intra- and inter-hospital spread and possible intercontinental circulation of CPE isolates.

## Introduction

Carbapenems, a class of β-lactam antibiotics with wide activity, are often the antimicrobials of last resort to treat infections associated to extended-spectrum β-lactamase (ESBL)- or plasmid-mediated AmpC (PMAβ)-producing *Enterobacteriaceae* isolates (Papp-Wallace et al., 2011; Rodríguez-Baño et al., 2018). Unfortunately, carbapenem non-susceptible *Enterobacteriaceae* (CNSE) have been reported worldwide mainly because of the acquisition of carbapenemase-encoding genes (Potter et al., 2016; Codjoe and Donkor, 2018). Since the first description of a carbapenemase-producing *Enterobacteriaceae* (CPE) in Europe in the 1990s, a large variety of carbapenemases has been identified in each of the four Ambler molecular classes, mainly the KPC-type (class A), VIM-, IMP-, and NDM-types (class B), and OXA-48-type (class D) (Grundmann et al., 2017; Logan and Weinstein, 2017). CPE isolates are usually resistant to many other β-lactam and non-β-lactam antibiotics, leading to multi-resistant isolates.

In Portugal, the epidemiological stage for the spread of CPE increased from sporadic isolates or single hospital clones, from April 2010 to February 2013, to sporadic hospital outbreaks later (Albiger et al., 2015; Manageiro et al., 2015b,c). Here we report data from a 6-month prevalence study performed under the European Survey on Carbapenemase-Producing *Enterobacteriaceae* (EuSCAPE) with the collaboration of different Portuguese Laboratories.

## Materials and Methods

### Bacterial Isolation, Antibiotic Susceptibility, and Molecular Characterization

This study included a total of 104 clinical isolates (94 *Klebsiella pneumoniae* and 10 *Escherichia coli*) collected from November 2013 to April 2014 in 10 Portuguese hospitals. The first ten consecutive and non-replicated CNSE isolates obtained during this period, in each hospital, from blood, lower respiratory tract secretions, urine, puncture fluids, and wound secretions, of single patients, were sent to the National Reference Laboratory, in Lisbon, and were considered. Successive carbapenem-susceptible isolates of the same species were also preserved as controls whenever possible, accordingly to EuSCAPE protocol (Grundmann et al., 2017). Overall, 67 CNSE (61 *K. pneumoniae* and 6 *E. coli*) and 37 controls (33 *K. pneumoniae* and 4 *E. coli*) were analyzed.

In the context of the EuSCAPE study, all data were anonymized and collected in accordance with the European Parliament and Council decisions on the epidemiological surveillance and control of communicable disease in the European Community (Eur-Lex-31998D2119, 1998; Eur-Lex-32000D0096, 2000).

### Antibiotic Susceptibility and Molecular Characterization of Antimicrobial Resistance

Antimicrobial susceptibility was performed by disk diffusion method for 15 antibiotics (Table 1), and by broth microdilution method for tigecycline and colistin, using EUCAST guidelines^1^. Clinical isolates with resistance or with decreased susceptibility to ertapenem were considered presumptively CPE. Isolates were considered multidrug resistant when presenting reduced susceptibility to three or more structurally unrelated antibiotics.

**Table 1:** Antimicrobial susceptibility of 67 (61 *K. pneumoniae* and 6 *E. coli*) CNSE and 37 (33 *K. pneumoniae* and 4 *E. coli*) control isolates.

Antibiotic	*K. pneumoniae*				*E. coli*			
	Control (*n* = 33)		CNSE (*n* = 61)		Control (*n* = 4)		CNSE (*n* = 6)	
	IR (%)	S (%)	IR (%)	S (%)	IR (%)	S (%)	IR (%)	S (%)
Ampicillin	100	0	100	0	100	0	100	0
Amoxicillin/Clavulanate	30	70	89	11	25	75	100	0
Piperacillin/Tazobactam	58	42	98	2	25	75	100	0
Cefotaxime	30	70	92	8	25	75	100	0
Ceftazidime	36	64	95	5	25	75	100	0
Cefepime	36	64	90	10	25	75	100	0
Aztreonam	30	70	92	8	25	75	100	0
Imipenem	0	100	59	41	0	100	100	0
Meropenem	0	100	64	36	0	100	100	0
Ertapenem	0	100	100	0	0	100	100	0
Ciprofloxacin	36	64	69	31	25	75	100	0
Gentamicin	21	79	64	36	50	50	83	17
Tobramycin	33	67	74	26	50	50	83	17
Amikacin	0	100	18	82	0	100	17	83
SXT	33	67	90	10	50	50	67	33
**Colistin^∗^**	6	94	11	89	0	100	0	100
MIC_50_	1		1		1		1
MIC_90_	2		4		2		2
**Tigecycline^∗^**	39	61	56	44	0	100	33	67
MIC_50_	1		2		0.5		0.5
MIC_90_	4		4		1		4

*^∗^Microdilution method.*

PCR and sequencing were applied to detect and identify the main CPE (*bla*_KPC_ and *bla*_GES_ from class A; *bla*_IMP_, *bla*_V IM_, and *bla*_NDM_ from class B; and *bla*_OXA–48_ from class D)-, ESBL (*bla*_TEM_, *bla*_SHV_, *bla*_OXA_, *bla*_CTX–M_) – and PMAβ (*bla*_CMY_, *bla*_MOX_, *bla*_FOX_, *bla*_LAT_, *bla*_ACT_, *bla*_MIR_, *bla*_DHA_, *bla*_MOR_, *bla*_ACC_)-encoding genes, as previously described (Manageiro et al., 2015b). Plasmid-mediated colistin resistance-encoding genes (*mcr*-type) were also investigated (Manageiro et al., 2017).

### Transfer Experiments

Transferability of *bla*_KPC–21_ from *E. coli* UR19829 was performed by broth mating out assays using sodium azide-resistant *E. coli* J53 as a recipient strain, and by transformation, as previously described (Manageiro et al., 2015b, 2017).

### Molecular Typing

Clonal relatedness of 67 CNSE isolates was investigated by pulsed-field gel electrophoresis (PFGE) as previously described (Manageiro et al., 2017). Genetic diversity of the *K. pneumoniae* (*n* = 10, i.e., 1 representative of each PFGE cluster) and *E. coli* (*n* = 10) isolates was investigated by multilocus sequence typing (MLST) (Manageiro et al., 2015b). *E. coli* sequence type (ST) subclones were also analyzed on the basis of the *E. coli* *fimH* gene (Manageiro et al., 2015a).

### Genomic Characterization of KPC-21-Producing *E. coli*

KPC-21-producing *E. coli* was genotypically characterized by whole-genome sequencing (WGS) as previously described (Manageiro et al., 2017). The assembled contigs were analyzed and studied for the presence of antibiotic resistance- and virulence-encoding genes, multi-locus sequence types, *fim* type, serotype, plasmid replicon types, and insertion sequences (ISs) using bioinformatics tools from the Center for Genomic Epidemiology^2^ and ISsaga (Varani et al., 2011).

The pUR19829-KPC21 plasmid structure was constructed based on the genetic organization of the closest plasmid sequences obtained by BLASTn, provided by NCBI^3^, followed by contig neighbor’s prediction from assembly information.

### Statistical Analysis

OpenEpi software, version 3.01 was used for statistical analysis (Sullivan et al., 2009). Fisher exact test was used to assess differences in clinical and epidemiological risk factors for control and CNSE-carrying patients. One-tailed *P* values of ≤0.05 were considered to be statistically significant. Associations were determined by calculation of odds ratios with 95% confidence intervals.

### Nucleotide Sequence Accession Number

The new *bla*_KPC–21_ nucleotide sequence was submitted to the NCBI GenBank Database with accession number NG_049254 and the complete plasmid sequence of pUR19829-KPC21 with accession number MH133192.

## Results and Discussion

During the study period, 67 isolates (61 *K. pneumoniae* and 6 *E. coli*) CNSE were identified in nine of the 10 Hospital Laboratories, with a non-susceptibility rate for meropenem and imipenem of 64 and 59%, respectively, for *K. pneumoniae*, and of 100% for *E. coli*. As expected, when compared with the control isolates, CNSE presented higher level of non-susceptibility to all antibiotic classes tested (Table 1). Colistin and tigecycline MIC_50_ values for CNSE were similar than those obtained for control isolates. Eleven out of the 104 (16.3%) isolates were colistin resistant, without the presence of the plasmid-mediated *mcr-1* or *mcr-2* gene. However, MCR-1 determinant was already identified in different reservoirs in Portugal, such as vegetables, animals and humans (Jones-Dias et al., 2016; Beyrouthy et al., 2017; Kieffer et al., 2017).

Thirty-eight (56.7%) isolates (36 *K. pneumoniae*, 2 *E. coli*) were confirmed to be CPE; we identified 36 *bla*_KPC–type_ (including one new variant: *bla*_KPC–21_), 1 *bla*_GES–5_, and 1 *bla*_GES–6_ plus *bla*_KPC–3_, alone or in combination with other *bla* genes (Supplementary Figure S1). The remaining 29 isolates were non-susceptible to carbapenems possibly due to porins deficiency with association of PMAβ (CMY-2 and DHA-1) and/or ESBL (mainly CTX-M-15) production (Martínez-Martínez, 2008).

**FIGURE 1:**
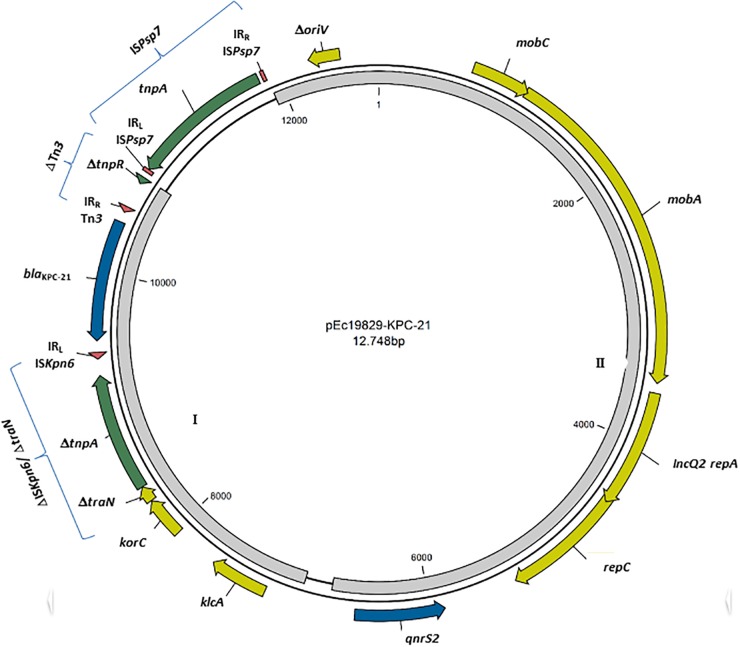
Schematic representation of KPC-21-harboring plasmid (pUR19829-KPC21). Genes are denoted by arrows. Blue, antibiotic resistance genes; Green, mobile genetic elements; Yellow, plasmid mobilization genes and replication origin. Right and left inverted repeats (IR_R_ and IR_L_) are indicated as red triangles. Gray regions 1: 99.97% of identity with KX756453, and Gray region 2: >99.9% sequence identity to KT896499.

The new *bla*_KPC–21_ gene differed from *bla*_KPC–2_ by one point mutation that leads to the amino acid substitution Trp105Arg; this position is involved in the binding and maintaining of the KPC catalytic activity (Papp-Wallace et al., 2010). *In silico* typing revealed an KPC-21-producing *E. coli* belonging to ST131 clade C/H30, associated with the fimbriae-encoding *fimH* allele 30, which become the most dominant lineage since the 2000s (Nicolas-Chanoine et al., 2014; Pitout and DeVinney, 2017). Moreover, bioinformatics analysis of the KPC-21-producing *E. coli* identified this variant in a 12,748 bp length plasmid, with a mean coverage of 580-fold and GC content of 58.5% (Figure 1).

Dissemination of *bla*_KPC_ has been mainly supported by the horizontal transfer of Tn4401-type transposon, which harbors *tnpA* encoding a transposase, *tnpR* encoding resolvase, and two insertion sequence elements (ISKpn7 and ISKpn6) bracketing the *bla*_KPC_ gene (Cuzon et al., 2011). In this study, the *bla*_KPC–21_ gene was harbored on a non-Tn*4401* element (Chen et al., 2014), presenting upstream a partial IS*Kpn6* (ΔIS*Kpn6/*Δ*traN*) with the related left IR (IR_L_) and downstream truncated Tn3 transposon downstream (Figure 1). This region has 99.97% of identity with pKP1194a, a plasmid carried by a hospital-associated KPC-2-producing *K. pneumoniae* isolated in Brazil (Accession number KX756453) (Figure 1- gray region I); this suggest an intercontinental circulation of isolates and mobile genetic elements (MGE), and the consequent need of concerted actions against the spreading of antibiotic resistance, at a worldwide level. The pUR19829-KPC21 enclosed also an intact IS*Psp7* element, an insertion sequence from IS*30* family, firstly described in *Pseudomonas* spp. (Szuplewska et al., 2014). Furthermore, the pUR19829-KPC21 backbone contained a region coding for plasmid replication (IncQ2 *repA*, *repC*), and mobilization (*mobA, mobC*), showing >99.9% sequence identity to the corresponding regions of pKPSH169, a 7.7 Kbp *qnrS2*-harboring IncQ plasmid identified in municipal wastewater treatment facilities in Israel (Accession number KT896499) (Figure 1- gray region II); this similarity highlights the high level of promiscuity of isolates between clinical settings and environment, where both reservoirs play a role in the antibiotic resistance dissemination (Stokes and Gillings, 2011). However, the lack of conjugative elements or an *oriT* region, associated with the presence of a truncated *oriV* region (Figure 1) suggests that pUR19829-KPC21 plasmid is nonmobilizable (Smillie et al., 2010). This fact is corroborated by the absence of a successful plasmid conjugation or transformation.

The variables used in the evaluation of risk factors for infection or colonization of patients with CNSE or control isolates are present in Table 2. When compared to the 37 control strains, only ESBL-production and the patient admission at a hospital in the center of Portugal were significantly associated with CNSE isolates in the period of the study. In the era of ESBL-producing *Enterobacteriaceae*, the antibiotic regimens suggested for severe health-associated infections are necessarily based on carbapenems (Rodríguez-Baño et al., 2018). Unfortunately carbapenem use has being described as a risk factor for CPE acquisition, only preceded by the use of medical devices (van Loon et al., 2018). In addition, the present study attests that Portugal, during the period of the study, has a different CNSE geographical distribution with the center of Portugal significantly associated with carbapenem non-susceptibility. This fact corroborates previous studies which indicated that in Portugal, in 2015, only sporadic isolates or single hospital cases were described (Albiger et al., 2015).

**Table 2:** Evaluation of risk factors for patients with infections caused by carbapenem susceptible or CNSE bacteria.

Variables	CNS isolates (no.)	Control isolates (no.)	OR	95% CI	*P* value
**Region of patient admission**					
North	20	13	0.7874	0.3094–2.038	0.3666(*P*)
Center	11	1	6.977	0.9398–312.7	0.03098
LVT	36	23	0.7092	0.2848–1.726	0.2670(*P*)
**Patient age**					
≤18 years old	3	1	1.68	0.1294–91.01	0.5522
19–64	22	12	1.018	0.401–2.66	0.5727
≥65	37	18	1.299	0.5389–3.146	0.3305
Unknown	5	6	–	–	–
**Patient gender**					
Female	31	18	0.9098	0.3769–2.197	0.4885(*P*)
Male	35	14	1.787	0.7359–4.453	0.1142
Unknown	1	5	–	–	–
**Bacteria vs. host infection**					
Colonization	0	2	0	0.0–2.912	0.1243(*P*)
Infection	28	22	0.493	0.1989–1.194	0.06393(*P*)
Unknown	39	13	–	–	–
**Type of infection**					
Community Onset	26	15	0.9307	0.3804–2.305	0.5127(*P*)
Hospital Acquisition	31	11	2.022	0.8072–5.311	0.07451
Unknown	10	11	–	–	–
**Local of infection**					
Urinary tract infection	39	21	1.061	0.4334–2.574	0.5242
Blood infection	8	4	1.117	0.2737–5.463	0.5679
Pus production with bacteria	7	1	4.155	0.5004–194.5	0.1502
Lower respiratory tract infection	4	3	0.7219	0.1148–5.215	0.4825(*P*)
Other infections	8	2	2.356	0.4353–24	0.2365
Unknown	1	6	–	–	–
**ESBL production**					
Positive	37	8	4.406	1.66–12.87	0.0007772
Negative	30	29	0.227	0.0777–0.6024	0.0007772 (*P*)
**Total**	**67**	**76**			

*OR, odds ratios; CI, 95% confidence intervals. (P) indicates a one-tail P-value for protective or negative association. One-tailed P values of ≤ 0.05 are underlined. LVT, Lisbon and Tagus Valley.*

PFGE and MLST analysis showed an important diversity, with isolates belonging to distinct PFGE and STs (Supplementary Figure S1). With respect to *K. pneumoniae* (Supplementary Figure S1A), a total of 10 clusters and 25 unique PFGE profiles were generated using XbaI, indicating the that the circulating clones in that period were genetically diverse. However, carbapenemase-producing *K. pneumoniae* isolates were more clonal (six PFGE clusters including 69.4% of these isolates) than non-carbapenemase-producing *K. pneumoniae* (four PFGE clusters including 50.0% of these isolates). As shown in Supplementary Figure S1, both CNSE species showed intra- and inter-hospital spread (e.g., PFGE clusters KpI and KpIX), with some hospital-specific clones (e.g., PFGE clusters KpIV and KpVIII). However, as also showed in Spain in other EuSCAPE study (Esteban-Cantos et al., 2017), the carbapenem-non-susceptible *K. pneumoniae* population was more clonal than the carbapenem-susceptible population (data not shown). Ten different MLSTs were detected among carbapenemase-producing (ST14, ST15, ST45, ST231, and ST1513) and non-carbapenemase-producing (ST11, ST17, ST348, and ST395) *K. pneumoniae* isolates. At our knowledge, this is the first description of ST17, ST395, and ST1513 *K. pneumoniae* in Portugal (Manageiro et al., 2015b; Rodrigues et al., 2016; Vubil et al., 2017). Noteworthy, the GES-5 enzyme was detected in a ST231 *K. pneumoniae* isolate as previously reported in Portugal, but in the same hospital, which shows its capacity to maintain in clinical settings due to the selection pressure of this environment (Manageiro et al., 2015b). Furthermore, ST45 was recently the cause of a hospital-based outbreak caused by multidrug-resistant, KPC-3- and MCR-1-producing *K. pneumoniae* in Portugal (Mendes et al., 2018).

The high-risk clone carbapenemase-positive *K. pneumoniae* ST258 was not detected in this study or among clinical carbapenemase-producing *K. pneumoniae* isolates in Portugal (Manageiro et al., 2015b; Rodrigues et al., 2016; Vubil et al., 2017). However, concerning carbapenem-non-susceptible *E. coli*, besides the six different PFGE unique profiles, the isolates belongs all but two (ST405-*fimH27* and ST23-*fimH35*) to the ST131 clade C/H30 high-risk clone disseminated worldwide (Supplementary Figure S1B) (Woodford et al., 2011; Pitout and DeVinney, 2017). Noteworthy, this clone was already detected in Portugal among other antibiotic resistance reservoirs, such as in an *E. coli* strain isolated from a dolphin housed at a Zoo Park (Manageiro et al., 2015a); in dogs and cats with urinary tract infection (Marques et al., 2018); and in *E. coli* strains from wastewater and gulls (Varela et al., 2015). Again, this shows that clinical settings and different environmental compartments may be considered communicating vessels through which bacteria and resistance genes are able to flow (Stokes and Gillings, 2011).

Portugal was one of the EuSCAPE participating countries that presented higher proportions of KPC-positive *K. pneumoniae* (Grundmann et al., 2017). The percentage of carbapenem non-susceptible *K. pneumoniae* was low in invasive infections in the study period [2.4%, EARS-Net 2013]^4^. However, although the consumption of carbapenems has declined by 13.3% between 2012 and 2016 (PPCIRA, 2017), Portugal is reporting since 2013 a significant increasing trend of carbapenem non-susceptible *K. pneumoniae* [6.4%, EARS-Net 2016]^4^. The number of inter-institutional transmission is also increasing (Glasner et al., 2013; Albiger et al., 2015), being *K. pneumoniae* the principal cause of bacterial health-associated infections in Portugal, as in other European countries (ECDC, 2013). Of concern is the fact that KPC-producing organisms cause infections with high morbidity and mortality (Porreca et al., 2018; Rodríguez-Baño et al., 2018). These results reinforces that reducing antibiotic use alone is likely insufficient for reversing resistance (Lopatkin et al., 2017). We strongly believe that the chain of transmission of isolates and genes in clinical settings will be reduced or broken, especially with containment measures rigorously implemented and followed at local level.

## Members of the Network EuScape-Portugal

North region: C.H. São João E.P.E. (J.T. Guimarães), H. Braga (C. Iglesias), C.H. Póvoa de Varzim e Vila do Conde E.P.E. (F. Fonseca). Centre region: C.H.U. de Coimbra/Covões (H. Oliveira), C.H.U. de Coimbra/HUC (L. Boaventura), C.H. Médio Tejo E.P.E. (Ana Soares). Lisbon and Tagus Valley region: C.H. Oeste Norte E.P.E. (A. Vicente). H. Garcia de Orta E.P.E. (J. Diogo), C.H. Lisboa Ocidental E.P.E. (E. Gonçalves), C.H. Lisboa Central E.P.E. (M. Pinto).

## Author Contributions

VM designed the study, performed the molecular experiments, bioinformatics analysis, analyzed the data, and wrote the manuscript. RR, IBM, and EF performed the microbiological and molecular experiments, and analyzed the data. DAS and LV performed Illumina genome sequencing experiments. The Network EuSCAPE-Portugal participants acquired laboratory data. MC designed the study, wrote and reviewed the manuscript. All authors read and approved the final manuscript.

## Conflict of Interest Statement

The authors declare that the research was conducted in the absence of any commercial or financial relationships that could be construed as a potential conflict of interest.

## Footnotes

1 http://www.eucast.org/clinical_breakpoints/

2 https://cge.cbs.dtu.dk/services/

3 http://blast.ncbi.nlm.nih.gov/Blast.cgi

4 https://ecdc.europa.eu/

## Supplementary Material

FIGURE S1 Pulsed-field gel electrophoresis (PFGE) dendrogram and genetic relatedness of 61 *K. pneumoniae* **(A)** and 6 *E. coli* **(B)** CNSE isolates. Isolate number, hospital code, year of isolation, carbapenems antibiotic susceptibility, detected carbapenemases, extended-spectrum β-lactamases (ESBL), inhibitor resistant SHV (IRS), and plasmid-mediated AmpC (PMAβ), Multilocus sequence typing (MLST) for selected isolates and PFGE profile types are shown. These profiles, from 0001 to 0035, were defined as forming clusters KpI to KpX, for *K. pneumoniae*, and from 0001 to 0006 for *E. coli*. For *E. coli* isolates, *fim*-type is also shown.

## References

[B1] Albiger B. Glasner C. Struelens M. J. Grundmann H. Monnet D. L. and The European Survey of Carbapenemase-Producing Enterobacteriaceae (EuSCAPE) Working Group (2015). Carbapenemase-producing *Enterobacteriaceae* in Europe: assessment by national experts from 38 countries, May 2015. *Euro Surveill.* 20:30062. 10.2807/1560-7917.ES.2015.20.45.30062 26675038

[B2] Beyrouthy R. Robin F. Lessene A. Lacombat I. Dortet L. Naas T. (2017). MCR-1 and OXA-48 in vivo acquisition in KPC-producing *Escherichia coli* after colistin treatment. *Antimicrob. Agents Chemother.* 61:e02540-16. 10.1128/AAC.02540-16 28507112 PMC5527634

[B3] Chen L. Mathema B. Chavda K. D. DeLeo F. R. Bonomo R. A. Kreiswirth B. N. (2014). Carbapenemase-producing *Klebsiella pneumoniae*: molecular and genetic decoding. *Trends Microbiol.* 22 686–696. 10.1016/j.tim.2014.09.003 25304194 PMC4365952

[B4] Codjoe F. S. Donkor E. S. (2018). Carbapenem resistance: a review. *Med. Sci.* 6:1. 10.3390/medsci6010001 PMC5872158 29267233

[B5] Cuzon G. Naas T. Nordmann P. (2011). Functional characterization of Tn*4401*, a T*n3*-based transposon involved in *bla_KPC_* gene mobilization. *Antimicrob. Agents Chemother.* 55 5370–5373. 10.1128/AAC.05202-11 21844325 PMC3195030

[B6] ECDC (2013). *Point Prevalence Survey of Healthcare Associated Infections and Antimicrobial use in European Acute Care Hospitals, 2011-2012.* Stockholm: European Centre for Disease Prevention and Control.

[B7] Esteban-Cantos A. Aracil B. Bautista V. Ortega A. Lara N. Saez D. (2017). The carbapenemase-producing *Klebsiella pneumoniae* population is distinct and more clonal than the carbapenem-susceptible population. *Antimicrob. Agents Chemother.* 61:e02520-16. 10.1128/AAC.02520-16 28137818 PMC5365714

[B8] Eur-Lex-31998D2119 (1998). *Decision Number 2119/98/EC of the European Parliament and of the Council of 24 September 1998: Setting up a Network for the Epidemiological Surveillance and Control of Communicable Diseases in the Community.* Available at: http://data.europa.eu/eli/dec/1998/2119/oj

[B9] Eur-Lex-32000D0096 (2000). *Commission Decision Number 2000/96/EC of 22 December 1999 on the Communicable Diseases to be Progressively Covered by the Community Network Under Decision No 2119/98/EC of the European Parliament and of the Council.* Available at: https://eur-lex.europa.eu/eli/dec/2000/96(1)/oj

[B10] Glasner C. Albiger B. Buist G. Tambiæ Andraseviæ A. Canton R. Carmeli Y. (2013). Carbapenemase-producing Enterobacteriaceae in Europe: a survey among national experts from 39 countries, February 2013. *Euro Surveill.* 18:20525. 10.2807/1560-7917.ES2013.18.28.20525 23870096

[B11] Grundmann H. Glasner C. Albiger B. Aanensen D. M. Tomlinson C. T. Andraseviæ A. T. (2017). Occurrence of carbapenemase-producing *Klebsiella pneumoniae* and *Escherichia coli* in the European survey of carbapenemase-producing Enterobacteriaceae (EuSCAPE): a prospective, multinational study. *Lancet Infect. Dis.* 17 153–163. 10.1016/S1473-3099(16)30257-2 27866944

[B12] Jones-Dias D. Manageiro V. Ferreira E. Barreiro P. Vieira L. Moura I. B. (2016). Architecture of class 1,2, and 3 integrons from Gram-negative bacteria recovered among fruits and vegetables. *Front. Microbiol.* 7:1400. 10.3389/fmicb.2016.01400 27679611 PMC5020092

[B13] Kieffer N. Aires-de-Sousa M. Nordmann P. Poirel L. (2017). High rate of MCR-1-producing *Escherichia coli* and *Klebsiella pneumoniae* among pigs, Portugal. *Emerg. Infect. Dis.* 23 2023–2029. 10.3201/eid2312.170883 29148380 PMC5708242

[B14] Logan L. K. Weinstein R. A. (2017). The epidemiology of carbapenem-resistant Enterobacteriaceae: the impact and evolution of a global menace. *J. Infect. Dis.* 215 S28–S36. 10.1093/infdis/jiw282 28375512 PMC5853342

[B15] Lopatkin A. J. Meredith H. R. Srimani J. K. Pfeiffer C. Durrett R. You L. (2017). Persistence and reversal of plasmid-mediated antibiotic resistance. *Nat. Commun.* 8:1689. 10.1038/s41467-017-01532-1 29162798 PMC5698434

[B16] Manageiro V. Clemente L. Graça R. Correia I. Albuquerque T. Ferreira E. (2017). New insights into resistance to colistin and third-generation cephalosporins of *Escherichia coli* in poultry, Portugal: novel *bla*_CTX-M-166_ and *bla*_ESAC_ genes. *Int. J. Food Microbiol.* 263 67–73. 10.1016/j.ijfoodmicro.2017.10.007 29031106

[B17] Manageiro V. Clemente L. Jones-Dias D. Albuquerque T. Ferreira E. Canica M. (2015a). CTX-M-15-producing *Escherichia coli* in Dolphin, Portugal. *Emerg. Infect. Dis.* 21 2249–2251. 10.3201/eid2112.141963 26583927 PMC4672440

[B18] Manageiro V. Ferreira E. Almeida J. Barbosa S. Simões C. Bonomo R. A. (2015b). Predominance of KPC-3 in a survey for carbapenemase-producing *Enterobacteriaceae* in Portugal. *Antimicrob. Agents Chemother.* 59 3588–3592. 10.1128/AAC.05065-14 25779587 PMC4432220

[B19] Manageiro V. Sampaio D. A. Pereira P. Rodrigues P. Vieira L. Palos C. (2015c). Draft genome sequence of the first NDM-1-producing *Providencia stuartii* isolated in Portugal. *Genome Announc.* 3:e01077-15. 10.1128/genomeA.01077-15 26404603 PMC4582579

[B20] Marques C. Belas A. Franco A. Aboim C. Gama L. T. Pomba C. (2018). Increase in antimicrobial resistance and emergence of major international high-risk clonal lineages in dogs and cats with urinary tract infection: 16 year retrospective study. *J. Antimicrob. Chemother.* 73 377–384. 10.1093/jac/dkx401 29136156 PMC5890753

[B21] Martínez-Martínez L. (2008). Extended-spectrum beta-lactamases and the permeability barrier. *Clin. Microbiol. Infect.* 14(Suppl. 1), 82–89. 10.1111/j.1469-0691.2007.01860.x 18154531

[B22] Mendes A. C. Novais Â Campos J. Rodrigues C. Santos C. Antunes P. (2018). *mcr-1* in carbapenemase-producing *Klebsiella pneumoniae* with hospitalized patients, Portugal, 2016–2017. *Emerg. Infect. Dis.* 24 762–766. 10.3201/eid2404.171787 29553327 PMC5875258

[B23] Nicolas-Chanoine M. H. Bertrand X. Madec J. Y. (2014). *Escherichia coli* ST131, an intriguing clonal group. *Clin. Microbiol. Rev.* 27 543–574. 10.1128/CMR.00125-13 24982321 PMC4135899

[B24] Papp-Wallace K. M. Endimiani A. Taracila M. A. Bonomo R. A. (2011). Carbapenems: past, present, and future. *Antimicrob. Agents Chemother.* 55 4943–4960. 10.1128/AAC.00296-11 21859938 PMC3195018

[B25] Papp-Wallace K. M. Taracila M. Wallace C. J. Hujer K. M. Bethel C. R. Hornick J. M. (2010). Elucidating the role of Trp105 in the KPC-2 β-lactamase. *Protein Sci.* 19 1714–1727. 10.1002/pro.454 20662006 PMC2975135

[B26] Pitout J. D. D. DeVinney R. (2017). *Escherichia coli* ST131: a multidrug-resistant clone primed for global domination. *F1000Res.* 6:F1000 Faculty Rev-195. 10.12688/f1000research.10609.1 28344773 PMC5333602

[B27] Porreca A. M. Sullivan K. V. Gallagher J. C. (2018). The Epidemiology, evolution, and treatment of KPC-producing organisms. *Curr. Infect. Dis. Rep.* 20:13. 10.1007/s11908-018-0617-x 29730830

[B28] Potter R. F. D’Souza A. W. Dantas G. (2016). The rapid spread of carbapenem-resistant Enterobacteriaceae. *Drug Resist. Updat.* 29 30–46. 10.1016/j.drup.2016.09.002 27912842 PMC5140036

[B29] PPCIRA (2017). *Programa de Prevenção e Controlo de Infeções e de Resistência aos Antimicrobianos 2017.* Lisboa: Direção-Geral da Saúde.

[B30] Rodrigues C. Bavloviè J. Machado E. Amorim J. Peixe L. and Novais, Â (2016). KPC-3-producing *Klebsiella pneumoniae* in Portugal linked to previously circulating non-CG258 lineages and uncommon genetic platforms (Tn4401d-IncFIA and Tn4401d-IncN). *Front. Microbiol.* 7:1000. 10.3389/fmicb.2016.01000 27446040 PMC4923139

[B31] Rodríguez-Baño J. Gutiérrez-Gutiérrez B. Machuca I. Pascual A. (2018). Treatment of infections caused by extended-spectrum-beta-lactamase-, AmpC-, and carbapenemase-producing *Enterobacteriaceae*. *Clin. Microbiol. Rev.* 31:e00079-17. 10.1128/CMR.00079-17 29444952 PMC5967687

[B32] Smillie C. Garcillán-Barcia M. P. Francia M. V. Rocha E. P. de la Cruz F. (2010). Mobility of plasmids. *Microbiol. Mol. Biol. Rev.* 74 434–452. 10.1128/MMBR.00020-10 20805406 PMC2937521

[B33] Stokes H. W. Gillings M. R. (2011). Gene flow, mobile genetic elements and the recruitment of antibiotic resistance genes into Gram-negative pathogens. *FEMS Microbiol. Rev.* 35 790–819. 10.1111/j.1574-6976.2011.00273.x 21517914

[B34] Sullivan K. M. Dean A. Soe M. M. (2009). OpenEpi: a web-based epidemiologic and statistical calculator for public health. *Public Health Rep.* 124 471–474. 10.1177/003335490912400320 19445426 PMC2663701

[B35] Szuplewska M. Ludwiczak M. Lyzwa K. Czarnecki J. Bartosik D. (2014). Mobility and generation of mosaic non-autonomous transposons by Tn3-derived inverted-repeat miniature elements (TIMEs). *PLoS One* 9:e105010. 10.1371/journal.pone.0105010 25121765 PMC4133298

[B36] van Loon K. Voor In ‘t Holt A. F. Vos M. C. (2018). A systematic review and meta-analyses of the clinical epidemiology of carbapenem-resistant *Enterobacteriaceae*. *Antimicrob. Agents Chemother.* 62:e01730-17. 10.1128/AAC.01730-17 29038269 PMC5740327

[B37] Varani A. Siguier P. Gourbeyre E. Charneau V. Chandler M. (2011). ISsaga is an ensemble of web-based methods for high throughput identification and semi-automatic annotation of insertion sequences in prokaryotic genomes. *Genome Biol.* 12:R30. 10.1186/gb-2011-12-3-r30 21443786 PMC3129680

[B38] Varela A. R. Manageiro V. Ferreira E. Guimarães M. A. da Costa P. M. Canica M. (2015). Molecular evidence of the close relatedness of clinical, gull and wastewater isolates of quinolone-resistant *Escherichia coli*. *J. Glob. Antimicrob. Resist.* 3 286–289. 10.1016/j.jgar.2015.07.008 27842875

[B39] Vubil D. Figueiredo R. Reis T. Canha C. Boaventura L. Da Silva G. J. (2017). Outbreak of KPC-3-producing ST15 and ST348 *Klebsiella pneumoniae* in a Portuguese hospital. *Epidemiol. Infect.* 145 595–599. 10.1017/S0950268816002442 27788691 PMC9507641

[B40] Woodford N. Turton J. F. Livermore D. M. (2011). Multiresistant Gram-negative bacteria: the role of high-risk clones in the dissemination of antibiotic resistance. *FEMS Microbiol. Rev.* 35 736–755. 10.1111/j.1574-6976.2011.00268.x 21303394

