# Supplementary figures and images for: Molecular Epidemiology and Risk Factors of Carbapenemase-Producing Enterobacteriaceae Isolates in Portuguese Hospitals: Results From European Survey on Carbapenemase-Producing Enterobacteriaceae (EuSCAPE)
Source: Front Microbiol. 2018 Nov 27;9:2834. doi: 10.3389/fmicb.2018.02834 (PMC6277554; doi:10.3389/fmicb.2018.02834)

Fig. S1A

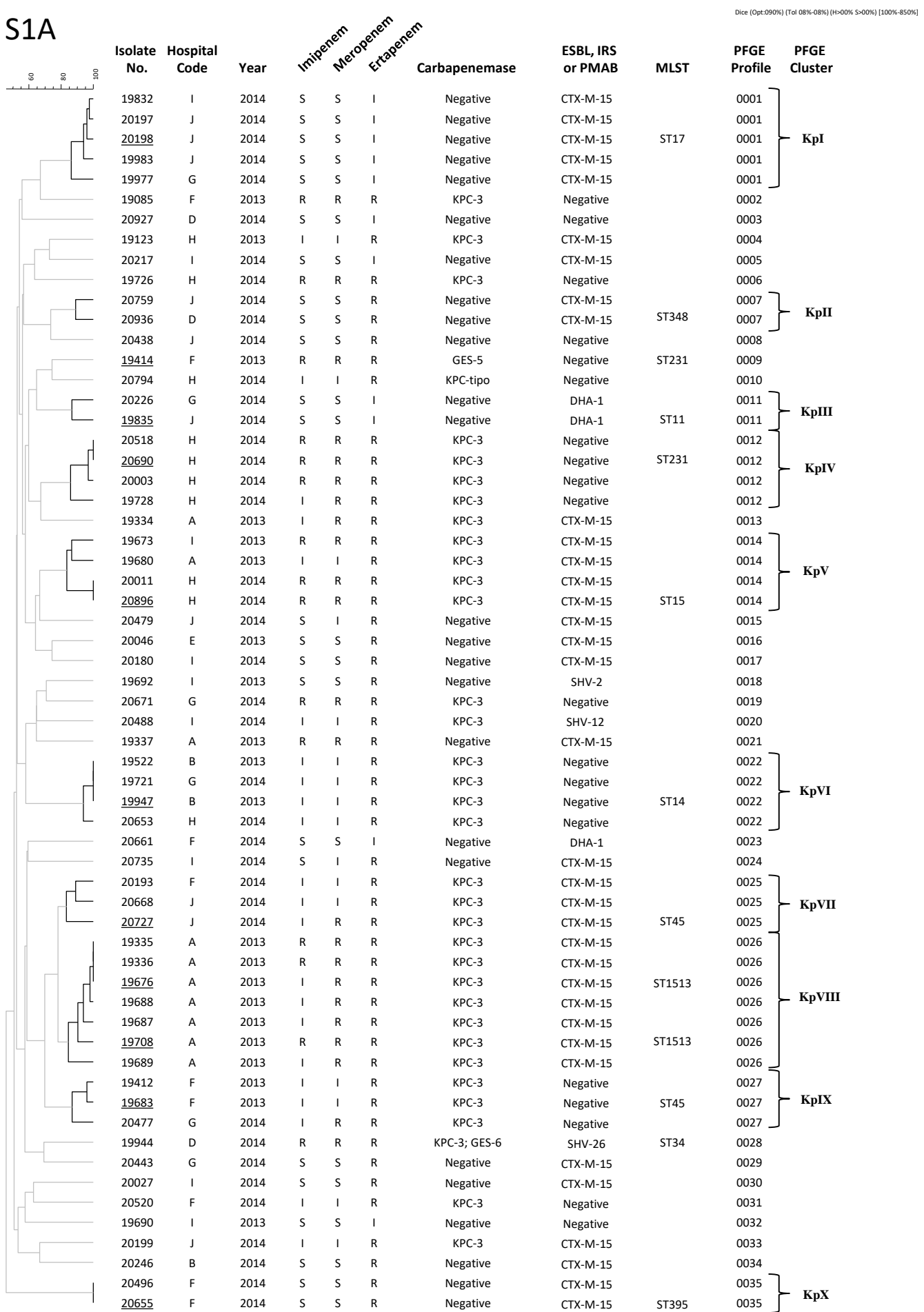

Fig. S1B

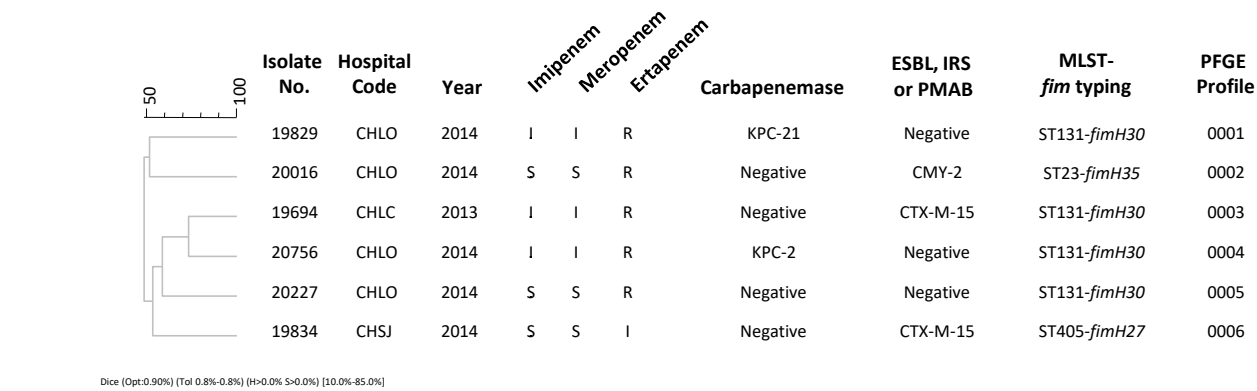

Supplement: FIGURE S1 — Pulsed-field gel electrophoresis (PFGE) dendrogram and genetic relatedness of 61 K. pneumoniae (A) and 6 E. coli (B) CNSE isolates. Isolate number, hospital code, year of isolation, carbapenems antibiotic susceptibility, detected carbapenemases, extended-spectrum β-lactamases (ESBL), inhibitor resistant SHV (IRS), and plasmid-mediated AmpC (PMAβ), Multilocus sequence typing (MLST) for selected isolates and PFGE profile types are shown. These profiles, from 0001 to 0035, were defined as forming clusters KpI to KpX, for K. pneumoniae, and from 0001 to 0006 for E. coli. For E. coli isolates, fim-type is also shown. [file Data_Sheet_1.PDF]
